# Implementing outcome-based education and student-centered learning in Afghan public universities: the current practices and challenges

**DOI:** 10.1016/j.heliyon.2021.e07076

**Published:** 2021-05-21

**Authors:** Rahmatullah Katawazai

**Affiliations:** Kandahar University, Afghanistan

**Keywords:** Attitudes, Lecturers, Outcome-based education, Student-centered learning, Public universities, Afghanistan

## Abstract

Outcome-based Education is currently of much potential in the global educational landscape. In recent years, implementing outcome-based education and student-centered learning have also been prioritized at the policy level of the Ministry of Higher Education of Afghanistan. Thus, the current study aimed to investigate the attitudes of Afghan lecturers towards the recent paradigm shift in the educational system of Afghanistan. The researcher employed a mixed-method approach to collect both quantitative and qualitative data. The researcher used a questionnaire responded by 120 lecturers and interviewed 7 outcome-based education experts and Afghan lecturers. The quantitative findings reveal that teachers have positive attitudes towards outcome-based education, and they are ready and willing to implement the approach by mentioning some key challenges. However, the results also show that implementing this approach is currently at a low level of practice. The qualitative findings reveal that; content-based curriculum, policies of teaching, learning, and assessment, the lack of basic infrastructure and info-structure, lack of facilities, and teachers’ workload are among the key challenges towards implementing this approach. The results will help the Ministry of Higher Education and Higher Education Development Program to develop applicable policies and to emphasize this recent paradigm shift in the future within addressing the key challenges to ensure the efficacy during implementation. It will also help lecturers to have an overall picture of outcome-based education and student-centered learning. Likewise, it will open a window for researchers to investigate this potential topic in more in-depth details in the higher education system of Afghanistan.

## 1 Introduction

Afghanistan is one of the conflict-affected countries for the last four decades. The country has had continuous conflicts in internal and external matters particularly in ethnic, tribal, and religious issues internally and the interference of the neighboring, regional, and some of the global countries have further increased the violence and kept the country to be far away from the ongoing developments of 21^st^ century in many fields, particularly, in terms of quality education. For this reason, not only politically, economically, and socially, the country suffered through many of the educational uncertainties as well including a very low rate of literacy and education in the country. For example, during the Taliban regime, only 1 million children were enrolled in schools where the higher percentage were boys, and most of the schools were supported by Non-governmental Organizations (NGOs) during such conditions (Strand, 2015). After 2001, the new government (the Islamic Republic of Afghanistan) took the responsibility to provide quality services in the field of education, and as Strand (2015) states, the current number of students is increased to 8.35 million students, where (39%) are girls but there are still some critical issues of corruption, infrastructure, school facilities, the appointment of teachers and overall the low-quality services of education in the country. Mostly, the main reason for rising such serious issues is the lack of security and weak governance that prevented providing quality services to most of the parts of the country, particularly, those regions located in war zones. However, the Ministry of Education (MoE) is committed to doing well and some of the improvements regarding the educational policies, curriculum framework for primary and secondary education and providing services about the infrastructure, the increasing number of teachers and students are among the hopeful matters MoE achieved after 2001 with the support of international donors and NGOs.

Within the improvements in the field of primary and secondary education, the increase of tertiary education in higher education institutions and governmental and private universities in the capital city, and other provinces of Afghanistan are currently in the expectant points and the demand for enrolling into higher education public and private institutions is increasing while compared with the era before 2001 (Berger and Thoma, 2015). It means that like any other aspect of life in Afghanistan, higher education is also significantly developing in the last two decades and some steps are taken regarding the policy frameworks and some other procedures (e.g. institutional autonomy in the private sector) as well (Berger and Thoma, 2015). However, too, in providing quality services including the lack of higher academic level of lecturers, lack of professional teachers in different departments, lack of infrastructure, lack of standard curriculum, lack of teaching and learning facilities and the limited use of ICTs (Information and Communication Technologies) in the higher education system of Afghanistan are amongst the serious obstacles that are still remaining challenging in tertiary education (MoHE, 2016).

The Ministry of Higher Education (MoHE) and Higher Education Development Program (HEDP) together, with the financial support of some NGOs and World Bank, struggle to provide quality services in the field of quality assurance, research, curriculum, and most importantly, changing the traditional system of higher education into the recently introduced educational system of Outcome-Based Education and Student-centered Learning (OBE-SCL) in all of the public universities of Afghanistan (MoHE, 2016). It means that today, implementing such kinds of systems is one of MoHE's thrust in the Afghan public universities. Hence, the focus of MoHE is that the public universities should be ‘dominant higher education institutions’ in the country for the purpose that the qualified students should make it as their first choice for pursuing their tertiary education and the public universities should be viewed as the high-status learning institutions for all of the Afghan learners (Berger and Thoma, 2015). In a report together by MoHE and HEDP (2016), the vision for the upcoming 5 years states that “To modernize teaching and learning in higher education of Afghanistan by promoting and institutionalizing outcome-based education and student-centered learning.” For achieving this goal, the Ministry of Higher Education of Afghanistan (MoHE) and HEDP started working on implementing this model in 2016 with the cooperation of Universiti Teknologi Malaysia (UTM), recognized as a good center of OBE-SCL in Malaysia. Although public universities offer free education to the Afghan learners (within the primary and secondary education free for all in all the Afghan public schools), MoHE views implementing OBE-SCL at Afghan public universities as not only one of the needs towards moving globalization, but a requirement of the ongoing 21st-century as well which is, as it is stated in the plan of MoHE (2016), one of the target points to be achieved for the modernizing higher education system in Afghanistan. Shifting from the traditional educational system into the new OBE-SCL will not only affect the curriculum but teaching and learning practices too. Considering the lack of tertiary level teachers' expertise and the traditional manner of students towards such kinds of SCL classroom activities and the lack of quality textbooks in the primary and secondary levels education are remaining as the big obstacles towards implementations of OBE-SCL in the Afghan context (Ahmadzai et al., 2019; Katawazai et al., 2019). Further, MoHE needs to have fulfilled some of the institutional tasks regarding the educational philosophy and practices, but the attainment of the specified outcomes, fully adapting some of the assessment procedures and criteria along with the curriculum changes based on these pre-specified outcomes is required. However, within the above concerns, MoHE and HEDP strive to follow and achieve their vision of changing the traditional educational system with a more modernized and globalized one in the public higher education institutions.

While discussing the terms of OBE-SCL, it has been nominated with many other names as well from the earlier decades such as Transformation Education, Competency-Based Education, Performance-Based Education, and Results-Based Education (Gieseen-Hood, 1999; Harden, 2002). Currently, it is one of the accepted and successful educational systems in many countries including the U.S., U.K., New Zealand, Malaysia, Australia, South Africa, and the Philippines, and totally it is currently embraced in over 47 countries around the globe (Baguio, 2019; Midraj, 2018; Rao, 2015; Tam, 2014). OBE-SCL has been viewed as one of the successful approaches that enable students’ active participation in classroom activities which closely links its theoretical foundations with the contemporary approaches as; active learning, constructivism, discovery-learning, student-centered learning, and ensure educational institutions easily reach their pre-determined aims, goals, and objectives, where all the teaching and learning activities are defined in clear statements in the curriculum (Baguio, 2019; Harden et al., 1999; Midraj, 2018).

The global trends in higher education from the traditional (teacher-centered) model into the modern (student-centered) model is one of the main shifts that is considered as one of the vital changes in many countries worldwide (Tam, 2014). The current requirements of the 21st-century are not only bounded with the economics, politics, and socio-economic matters, but it mostly emphasizes educational paradigms including teaching and learning activities and assessment in every discipline and specific personal skills. It means that individual capability and skillfulness are nowadays considered as one of the vital criteria that a graduate needs to acquire to be employed and accepted in the market. For this reason, the educational paradigm is being changed in many countries for the purpose to be aligned with the current requirements of this century. Currently, OBE-SCL is one of the main thrusts of the Ministry of Higher Education in Afghanistan to shift from the traditional (content-based) education to the recently introduced educational approach (OBE-SCL) to be implemented in all of the Afghan Public Universities. This shift has impacted the Afghan higher education system and the attitudes of both lecturers and learners to be able to adapt themselves to the changes and the requirements that OBE-SCL needs. As in a recent study, Alimyar (2020) also stated that the adaptation to the outcome-based education has impacted the attitudes of the lecturers positively and even they felt responsible while implementing this approach in their classrooms. Therefore, this created an opportunity for the Afghan public institutions to make applicable policies in order to shift from the classical ways of teaching and learning and to use new procedures instead.

Although the demand for OBE-SCL is increasing in Afghanistan, particularly in the Ministry of Higher Education and public universities, the implementation of OBE-SCL is still in its early stages in this country and much more steps are needed for the successful adaptation of this educational system in tertiary level. However, it needs to investigate the attitudes of faculty members, the current practices, and the extent of the new system within the challenges that the Afghan public university lecturers have been faced during the process of utilizing OBE-SCL in their classrooms.

The purpose of the current study is to investigate the attitudes of Afghan lecturers and the perspectives of OBE-SCL experts regarding the new educational system and to identify the current classroom practices in the public universities of Afghanistan within the challenges, if any, in the process of shifting from the classical model of teaching into the modernized one. The need for the study is that as the educational system is recently changing from the content-based (traditional) to the outcome-based (modern) education, so the Afghan policymakers, curriculum developers, educators, and staff should be aware of all the possible challenges that influence the successful implementation of OBE-SCL and to keep the recommendations that are helpful regarding the adaptation of all the involved aspects in the process for utilizing OBE-SCL in the Afghan context. Moreover, the perspectives of Afghan lecturers can be considered as among the challenges that will make the successful implementation of the process problematic. It means that as the new system will focus on students' engagement rather than teachers’, so it will lead to the main shift in the teaching and learning activities as well and teachers will need to be responsibly ready for such a new shift of education. Hence, understanding, knowledge, and the adjustment of Afghan lecturers will help the successful application of OBE-SCL and where they need to know the phenomenon.

The focus of the current study is to look into this recent educational trend in the Afghan context and the current study is specifically looking to find out appropriate answers for the following research questions:

1) *What are the attitudes of Afghan lecturers towards outcome-based education and student-centered learning?*
2) *What is the extent of implementing outcome-based education and student-centered learning at Afghan public universities?*
3) *What are the challenges of implementing outcome-based education and student-centered learning in the Afghan context?*

## 2 Review of literature

### 2.1 The definitions of OBE-SCL in literature

Outcome-based education has been widely defined in literature differently. Spady (1988), who is considered as one of the leading advocates of outcome-based education, defined this approach as the design, development, and documenting of instruction in which goals and outcomes are pre-defined. He believes that the curriculum should be developed after an educational institution demonstrated the outcomes that they wish for their students to achieve after graduation. Gieseen-Hood (1999) defined OBE as ‘an educational approach’ where the focus of it is not only on what the students learn but also on how they learn. OBE-SCL is viewed as one of the systems in which the main focus is on the attainment of educational standards and learning outcomes (LOs) and the learners should be able to reach those (LOs) at the end of the learning experience where both (teacher) and (students) need to understand the intention of the teachers for teaching and the expectations of students to learn, understand and practically perform at the end of each course (Midraj, 2018; Tam, 2014). Tshai et al. (2014) stated that the focus of OBE-SCL is on two main components regarding the learners' achievement in an educational program. They stated program outcomes along with the program's educational outcomes as among the two main elements that are emphasized in this system. Furthermore, Gurukkal (2020) also opined and emphasized that for the better implementation of outcome-based education, academic institutions have to ensure that the outcomes have been determined appropriately and they are aligned with the curriculum contents. Similarly, Rao (2020) also emphasized that determining the types of outcomes must be not only achievable and measurable, but they should also be aligned with the three main domains (Affective domain, Cognitive domain, and Psycho-motor domain) of Bloom's Taxonomy.

Furthermore, Malan (2000) explains OBE as an eclectic educational philosophy, and believes that it is rooted in the previous approaches of teaching (for example, competency-based learning, active learning, mastery learning, criterion-referenced instruction) but framed in a new visionary educational system in which the needs of the learners have been pre-determined and pre-modified. Further point added by the researcher is that as a typical educational model, OBE allows stakeholders with a socio-constructivist base, regarding the input and the adaptation of the new demands and needs. Davis (2003) defined OBE as an educational approach, where decision making about curriculum should be made before the determination of the outcomes. It means that the outcomes existing about a course for the students should be displayed at the end of the course whether they have been achieved or not. In addition, Mitra and Gupta (2020) stated that the main purpose of outcome-based education is to prepare the graduates for ‘employability’ in the global market. It means that as the current employment market needs special professional, communicational, technical, problem-solving, and other skills, so academic institutions have a key responsibility to foster their graduates based on these demands of the global market. For this reason, this need of the century intended some countries to recheck and upgrade their educational systems on the basis of the requirements. As in Malaysia for instance, due to the unemployment matters in the Malaysian market, the Ministry of Higher Education of Malaysia planned to upgrade the traditional curriculum and as well as the ways of teaching, learning, and assessment. Therefore, Yusof et al. (2017) stated that the kind of curriculum that is best suited to overcome this situation is the OBE curriculum. It is because they defined OBE as an appropriate approach that shifts from the traditional content-based curriculum to the modern curriculum that can produce the types of graduates, who are ready for the economic change of the market and are skilled with the demands of the current global market.

### 2.2 The relationships of OBE-SCL with the earlier theories, curriculum models and paradigms

Deepak and Venishri (2018) view OBE as an adapted educational model where students' integrity is considered as one of the vital points for the educators of this model along with the tendency of individual positive motivation and in the process that positively influences their learning. OBE-SCL is rooted with the earlier theories and educational revolutions and it has been passed through many stages and movements in the field of education and shifted as the system and model that is currently in practice in most of the countries worldwide. Tyler in the early 1950s proposed an educational model (Achievement of Desired Outcomes) which Lewy (1977) views as probably one of the best-known models that is in use in the global educational institutions to measure the essence, quality, and excellence of an educational course or program. In Tyler's model, education is considered as one of the three-dimensional processes that include educational objectives, learning experiences, and examination of achievement to be inter-related with each other. Lewy (1977) stated that both (cognitive) and (affective) outcomes have received attention in Tyler's model. Malan (2000) views OBE to be closely rooted in Tyler's model of Achievement of Desired Outcomes and believes that both of them focus on some of the shared points. In this model, Tyler stated four questions (Tyler; in Arjun, 1998; 222) as follows:

1. What educational objectives should the school aim to achieve?
2. How does one select learning experiences that are likely to be useful in attaining these objectives?
3. How should learning experiences be organized for effective instruction?
4. How would the effectiveness of learning experiences be evaluated?

After Tyler's model, Wheeler (1969) created a sequential model of the curriculum based on Tyler's model which was considered as one of the main curriculum models for several decades (Arjun, 1998). Wheeler's model consisting of the following components: aims, goals and objectives, learning experiences, contents, integrating the learning experiences with contents and evaluation.

Besides, Bloom et al. (1956) work of developing taxonomies and educational objectives is also considered as one of the preliminary steps for the late OBE-SCL approach. Malan (2000) views Bloom's taxonomy, particularly, the cognitive domain, as one of the main requirements needed for OBE-SCL assessment. He further states that Bloom's standards were then used in the organization of the learning objectives and the development criteria to ensure that whether learners attained the expected learning outcomes or not. Competency-based education is another predecessor of OBE-SCL that was firstly introduced in America in the late 1960s in the reaction that what students learn at school is actually not applicable in their real-life situation (Malan, 2000). To overcome this problem, competency-based education was utilized in the process of teaching and learning. Van der Horst and McDonald (1997) (Cited by Malan, 2000) stated that this type of educational system consists of six critical components as follows:

1. Explicit learning outcomes with respect to the required skills and concomitant proficiency (standards for assessment)
2. A flexible time frame to master these skills
3. A variety of instructional activities to facilitate learning
4. Criterion-referenced testing of the required outcomes
5. Certification based on demonstrated learning outcomes
6. Adaptable programs to ensure optimum learner guidance

Malan (2000) believes that all of the above-mentioned components are considered in the OBE-SCL approach where both of them are focusing on learners’ accountability for the achievement of goal(s) in the process of learning.

Spady is known as a key advocate of the OBE and one of the theorists of this model. His definition of OBE is that it is a ‘comprehensive approach’ which is focusing on the success of each student and further adds that what students learn is more important than when and how they learn. Malan (2000) believes that the points made by Spady are sufficient enough in regard to the overall structure of OBE. Also, Biggs's (1996) theory of Constructive Alignment also seems to be closely linked with the OBE-SCL approach. Tam (2014) also explains that Biggs's theory of constructive alignment is one of the central rooted theories with the OBE-SCL where it is believed that the learning is the mean of constructing by the students during a particular course or program and it is the output of students' activities during the experiences that they get in classrooms and the focus is on the practicality at the end of a learning experience.

### 2.3 The benefits of OBE in the world literature

While reading the global literature, OBE-SCL is considered as one of the useful educational models that can foster students to be not only active learners but to be the best graduates as well in terms of their suitability with the demands of the global market. A study by McNeil et al. (2006) revealed that the implementation of OBE influenced the learning process positively with the attention given to the capabilities of graduates and overall the success of the whole program. A similar study was conducted by Wong and Cheung (2011) in Computer Science Education in Hong Kong. The results of their study show that OBE is a good educational system and can promote students' motivation and active learning. They claimed that implementing the OBE model of education will reach Hong Kong education to higher success, as seems in the results of the study. They not only recommend this educational model to be utilized in educational institutions in Hong Kong but emphasize on it as well that, as they believe, will lead the students to be trained as potential leaders in their society. Furthermore, the study of Kaliannan and Chandran (2012) in the Malaysian context shows that the implementation of OBE benefited the active learning of the students and helped faculty members in improving their teaching as well. They pointed out that the Quality Assurance Division along with the Ministry of Education in Malaysia decided to overcome the problem of the unemployment of the university graduates. For overcoming this challenge, they decided implementing OBE as a good solution for that to be able to foster students based on the pre-determined objectives needed for the market employment in Malaysia. They further added that during the time students receiving OBE, they were then able to measure their understanding in each subject and they could compare their efforts with their own achievement in a subject. Within students, lecturers also had the ability to measure students’ academic performance and to give them corrective and constructive feedback after that.

Rao (2015) explained that utilizing OBE will help not only students to benefit global opportunities, but stakeholders, parents, employers, and as well as, engineers and help governments to overcome the problem of unemployability of graduates. Similarly, Hegde (2015) stated that the implementation of the OBE approach for teaching and learning activities for the bachelor students of Telecommunication Engineering at BMS College of Engineering was effective enough. The researcher assessed students with two continuous internal assessment tests and a seminar. The findings of the study show that students of that particular department improved not only their professional skills and capabilities but generic skills and capabilities as well. Within the benefits that OBE-SCL has in the field of education, Deepak and Venishri (2018) view this model as one of the successful and perceived models for engineering education as well but they focus on the clearly specified outcomes that can be resulted from the encouragement of participation and collaboration.

Yuzainee et al. (2014) conducted a meta-analysis study in regard to outcome-based education. In total, they found and analyzed 30 empirical studies similar to their keywords through using different search engines like Google Scholar, ERIC, SCOPUS, and so on. Their main focus of the study was on the investigation of program outcomes and learning outcomes and their influences on the students' achievement in higher education institutions. The study shows a large effect size with an average of (1.574481) based on students’ achievement of implementing OBE. They stated that this model offered positive effects to both (teachers) and (learners) in terms of achieving program outcomes and learning outcomes and they emphasized OBE as an effective educational model to be used in higher education institutions. Similarly, Borsoto et al. (2014) conducted a study to find out the effectiveness of implementing OBE in the Engineering Department of an Asian University. The findings of their study show that the level of the respondents in regard to utilizing the model and its practices is very high and they viewed the model as one of the useful educational models for the academic achievement, attitudes, and instructions in that department. An (2014) also investigated the impact of OBE on students at an Asian University and the findings reveal that the model has very effective benefits for the students and the students who were instructed under this model were more productive after every single instruction. Similarly, Macatangay et al. (2016) studied the status and usefulness of the OBE model in one of the Asian Universities. Their findings indicate that the respondents of the study assessed the model as one of the successful models in that context.

In another study conducted by Isa et al. (2017) at one of the Malaysian public universities, Universiti Teknologi MARA, Shah Alam, in the Faculty of Civil Engineering, shows the same positive effects of the OBE-SCL model as stated in the earlier paragraphs. The findings reveal that by implementing this model, there seems an improvement in not only the technical skills of the students but other skills too as communication skills and the skills of being life-long learners. Researchers stated that because of the active learning activities, the students of that faculty benefited a lot and improved their multiple skills through receiving teaching and learning by utilizing the OBE approach. They further recommend the continuous assessment of the model at the university and emphasize its effectiveness to be ensured through quality assessment and monitoring.

A study was conducted by Senaratne and Gunarathne (2019) for the purpose to find out the implementation of OBE in a Sri Lankan University of Sri Jayewardenepura, Department of Accounting. Researchers stated that the Department of Accounting, with around 20 years of experience in internship and implementation of OBE shows that the model seems successful and it is because the graduates of the Department of Accounting benefited from it, particularly, in regard to the employment matters. Within, both teachers and students who went through some of the training in the OBE model claimed the success and the effectiveness of the model and they recommend it for others as well to use in the future methodologies of teaching and learning.

To sum up, by looking at some of the studies reported in the earlier paragraphs, it seems that OBE-SCL could be seen globally in different educational contexts and different disciplines as one of the effective educational models that have benefited learners in terms of academic performance, achievement, multiple technical and non-technical skills and, most importantly, of the employment and unemployment matters in the world market.

### 2.4 The challenges/disadvantages of OBE globally

The current literature shows that in most of the contexts of tertiary education, implementing OBE-SCL positively influenced the process of teaching and learning in different higher education institutions. To add more, studies show multiple positive influences of OBE-SCL on technical skills, non-technical skills, communication skills, and particularly, academic performance and employment-related skills. However, there are also some challenges and disadvantages that some research studies indicated the implementation of OBE-SCL in different contexts globally.

Jansen (1998) critically analyzed outcome-based education in the South African context. In this article, numerous negative points of OBE have been outlined in that particular context. For instance, the language and terms used in OBE are complex and confusing. With the complexity of terms that is problematic for teachers, these terms have turned into new meanings overtimes. For example, the term ‘outcome’ needs a very in-depth understanding to be defined and determined clearly. This research outlines the following points about OBE.

1. The relations of the OBE curriculum and its association with a particular society is problematic.
2. OBE is designed inappropriately for the South African educational system and context. The reason behind it is that the real situations inside the school, classroom management, the kind of teachers existing in the system are based on imperfect assumptions.
3. Some of the philosophical reasons in regard to OBE caused it to be questioned, particularly, in democratic schools.
4. The information related to OBE is not accessible to the teachers and the problem is in the majority in South Africa. It means that there is no systematic procedure where teachers can be able to improve their understanding of the concepts and principles of OBE and curriculum policy.
5. The outcomes identified for the South African context seem to be not as much clearly identified as it could be and there is no direction in OBE to avoid ill-defined outcomes to be more appropriate for a particular context.
6. The assessment policy seems to be placed on teachers. However, the large class size made the assessment procedures problematic for the teachers in the South African context and it seems that in reality, OBE seems against its success.
7. Selecting curriculum contents, changing or modifying the contents may be sometimes under political influences. For example, who makes the changes, where, and under which conditions?
8. The implementation of OBE requires all the teachers to be trained enough in terms of assessment procedures, classroom activities, and their management, additional time for managing the tasks, continuous monitoring of all the learning process, new forms of learning resources are among the key things, that will ensure the success of OBE.
9. As of other criticisms, OBE requires a radical revision of curriculum and namely the assessment system. In other words, some global experiences indicate that within the other principles achieved for the pre-determined outcomes, the criteria of assessment were still problematic and were not fully achievable.

To add more, while looking into the implementation of OBE-SCL in various educational levels, it seems that it has some drawbacks as well in different levels. For instance, in regard to the lower levels (primary and secondary), Davis (2003) outlined some of the points that are considered as the disadvantages in regard to OBE-SCL as follows:

1. The imposition of Constraints: outcome-based education is viewed as a constraint on children's education. The reason is that it is a constraint that a child should be kept independent where he/she goes and to be able to feel improvements that they can undergo.
2. The inappropriate emphasis of OBE-SCL on attitudes and values: the authors who are on the side of disagreement with this model believe that the outcomes defined are in favor of ill-defined values and skills which is a constraint towards learning the real attitudes and values that a learner needs to acquire and learn.
3. Inhibition of Learning by Discovery: McKernan (1993) (cited in Davis, 2003) argued that “education should be valued for its own sake and not because it led to a pre-identified outcome.” It means that it will be problematic for the learning that takes place through discovery and inquiry and it is because the outcomes have already been defined and the learners should go straight forward to follow those exact outcomes determined by an institution. However, it is claimed that this liberal notion of the OBE-SCL model is more appropriate in the arts and humanities.

Similarly, Eldeeb and Sharakumari (2013) stated in their article that within the benefits that the model of OBE has, there is some criticism on it as well in terms of being closely bound with outcomes which avoid the argument that education should be open-ended, not closed-ended, sometimes clearly determining the outcomes is problematic and as they stated, many scholars in the field of education oppose it as an inappropriate model for education. In a similar study conducted by Mogashoa (2013), the researcher found the implementation of the model problematic and stated that the lack of proper pieces of training, teachers’ understanding of this new system particularly in terms of aligning assessment procedures with the outcomes stated in the curriculum and the lack of adequate resources are needed for the successful implementation of OBE. The researcher also indicated that the teachers lacked to structure the classroom activities based on OBE, and it caused negative effects of the model in that context. In conclusion, as mentioned in the findings of the above studies, OBE-SCL resulted in some negative impacts rather than positive ones due to missing some pre-requisite facilities, enough training, and other requirements that are needed for the better results of it prior to its implementation.

## 3 Methodology

This research study employed a mixed-method approach in order to collect both numerical (quantitative) and descriptive (qualitative) data. The purpose of using this method is to make sure that the problem is investigated in in-depth details by collecting both quantitative and qualitative data. As Creswell (2014) also preferred this method for a more in-depth exploration of a phenomenon. The Study used convergent research design of mixed-method in order to find out appropriate responses for the research questions and to relate or compare both types of data. The researcher first collected quantitative data and then qualitative data and interpreted both types of data in the interpretation stage equally prioritized. Creswell (2014) stated that the researcher collects both numerical and descriptive data in this type of design and then merges the data and as well as using its results to understand the research problem.

### 3.1 Research process of the study

Based on the research problem exists of using traditional methods of teaching and learning in the Afghan context for a long time, the researcher conducted the current study to find out the attitudes of lecturers towards using outcome-based education models in their classrooms. Therefore, the researcher conducted a mixed-method study to investigate it in in-depth details. First, the researcher collected quantitative data by distributing a Google Form to 120 respondents lecturing in different public universities of Afghanistan. After that, the researcher interviewed 7 lecturers and OBE experts in order to find out clear answers regarding this method in the tertiary level education of Afghanistan. After data collection, the researcher analyzed quantitative and qualitative data to investigate the selected research topic thoroughly and to find out a suitable solution for the research problem.

### 3.2 Sample of research

The participants in the quantitative part were the lecturers at public universities of Afghanistan. A sampling size of 120 lecturers participated in this study. Based on Chuan and Penyelidikan (2006), this number of sampling size is manageable for quantitative research and the researcher can achieve the research objectives and research questions. The sampling method used for the quantitative data collection was the snowball sampling method where teachers from different public institutions were requested to forward and share the questionnaire with their other colleagues.

For the qualitative part, the researcher interviewed 7 lecturers and OBE experts in this study. The sampling method used for the qualitative part was the Quota sampling method. Mack et al. (2005) stated that in the qualitative research methods, the quota sampling method is one of the common types of sampling methods that qualitative researchers use. They further added that this type of sampling method is appropriate for the researchers who plan to include the type of participants with particular criteria or characteristics. Therefore, the researcher considered this type of sampling method suitable for qualitative data collection. For this reason, those lecturers who were OBE-SCL trainers and master trainers were interviewed to investigate the phenomenon thoroughly.

### 3.3 Instrument and procedures

For the quantitative part of the current study, the researcher used an eclectic questionnaire instrument which Creswell (2014) called an easy to use tool for collecting quantitative data. Section 1 consists of the items about the demographic details of the participants. The other sections consist of items about investigating different aspects of outcome-based education. This study employed two instruments (questionnaire and interviews) as primary tools. For the quantitative part, the researcher adapted the questions from (Baguio, 2019; Loreto, 2018; Ortega and Cruz, 2016). The quality and quantity of the questionnaires were already tested by the originators of the questionnaires. However, in the case of the current study, the researcher rechecked and retested the reliability and validity of the items and shared with experts and statisticians of SPSS. The Cronbach's Alpha level for the items is (.923) coefficient, which indicates that the internal consistency of the items for the current sample is very high and reasonable. Despite that, the questionnaire (From Section 2 up to Section 8) was also shared with several researchers and OBE-SCL experts from various universities of Afghanistan and one university of Malaysia.

For the qualitative part, semi-structured interview questions were prepared based on the OBE-SCL reports of MoHE, 2016, MoHE and HEDP 2016, and the eclectic questionnaire of the current study adapted from various researchers.

### 3.4 Data analysis

As this study employed a mixed-method research, the data were analyzed both statistically and qualitatively. For the quantitative part, the data were analyzed using Statistical Package for Social Sciences (SPSS 26.0) for finding out the descriptive statistics, frequency, tabulations, graphs, and charts. However, the qualitative data were analyzed using NVivo 12 for finding out the nodes in the qualitative data (interviews).

## 4 Findings and results

### 4.1 Quantitative results

The following sections reveal the quantitative findings of the current study collected through a questionnaire and analyzed in tabulation, frequencies, and descriptive statistics. Table 1 shows the demographic information of the respondents which include their age, gender, teaching experience, highest educational attainment, and the number of OBE-SCL training workshop that they participated.

**Table 1:** Demographic information of the respondents.

Age	Frequency	Percentage %
25–30 Years	57	47.5
31–35 Years	49	40.8
36–40 Years	11	9.2
>40 Years	3	2.5
*Total*	*120*	*100%*
**Gender**		
Male	106	88.3
Female	14	11.7
*Total*	*120*	*100%*
**Teaching Experience**		
1–5 Years	57	47.5
6–10 Years	51	42.5
11–25 Years	10	8.3
>15 Years	2	1.7
*Total*	*120*	*100%*
**Highest Educational Attainment**		
Bachelor	34	28.3
Master	81	67.5
PhD	5	4.2
*Total*	*120*	*100%*
**Participation in OBE-SCL Training**		
1 time only	47	39.2
2 times	18	15
3-5 times	8	6.6
>5 times	6	5
Not participated yet	41	34.2
*Total*	*120*	*100%*

As in Table 1 above, most of the respondents are young lecturers about (47.5%), 25–30 years of age, and (40.8%) are 31–35 years of age. Just a small number of respondents are 36–40 years of age and over which makes (11.7%) in total. In addition, (88.3%) of the respondents are male and it's because of the limited number of women are appointed in the higher education institutions in Afghanistan due to some social, religious, and cultural factors.

Strand (2015) conducted an investigation report on funding for education in Afghanistan. The report adds that due to certain country's social and religious obstacles, ‘only 21 percent of girls complete their primary education,’ and the report adds that girls' early marriage and the lack of female teachers are considered the country's most visible barriers towards women's education. Within these constraints, the Ministry of Higher Education aims to hire female teachers in tertiary education, but the number of educated women is not adequate to overcome the cultural sensitivity towards female students who wish to pursue their higher education. Similarly, as stated earlier that most of the respondents are youngers, so their teaching background in the higher education system is not too old and (47.5%) of the respondents taught for 1–5 years, (42.5%) of them are in 6–10 years of teaching experience. Among them, a very small number of respondents (10%) are having 11–15 years of teaching experience and above. Among them, the majority of the teachers having master's degrees which makes (67.5%) of all the respondents. Also, (28.3%) of the respondents are still having bachelor's degrees and a small number of the respondents are PhDs (4.2%).

In terms of participation in OBE-SCL related seminars and workshops, (54.2%) of the respondents had participated in the OBE-SCL training workshops once and twice until the time researcher conducted this study. Only a limited number of respondents (11.7%) had participated in such workshops 3–5 times and above. Unluckily, there is still a visible number of respondents (34.2%) who have not participated in OBE-SCL related training workshops yet. This large number of respondents have been remained untrained due to lack of enough training workshops inside and outside of public institutions.

Referring to Table 2 for identifying the attitudes of Afghan lecturers towards OBE-SCL which is being measured by asking 24 questions in terms of their knowledge, believes, feelings, their readiness for the new approach and acceptance level.

**Table 2:** Lecturers’ attitudes regarding knowledge, believes, feelings, readiness and acceptance of OBE-SCL

	Knowledge Level				
	Items	SA	A	D	SD
1.	I know where to start OBE-SCL in my class.	10 (8.3%)	50 (41.7%)	40 (33.3%)	20 (16.7%)
2.	I know how to facilitate an outcomes-based class.	17 (14.1%)	62 (51.7%)	30 (25.0%)	11 (9.2%)
3.	I can align the world of teaching with the world of working.	32 (26.6%)	41 (34.2%)	27 (22.5%)	20 (16.7%)
4.	I am equipped to make OBE-SCL classroom climate, providing cooperative, well-directed and purposeful activities.	15 (12.5%)	10 (8.3%)	51 (42.5%)	44 (36.7%)
5.	I have enough knowledge of the assessment techniques in OBE-SCL.	10 (8.4%)	31 (25.8%)	57 (47.5%)	22 (18.3%)
	*Total =*	*14%*	*32.33 %*	*34.17%*	*19.50%*
	**Beliefs**				
6.	I believe that OBE-SCL will improve students' academic achievements.	49 (40.8%)	54 (45.0%)	8 (6.7%)	9 (7.5%)
7.	I believe that OBE-SCL would require more contacts and communication with industry.	58 (48.3%)	44 (36.7%)	9 (7.5%)	9 (7.5%)
8.	I believe that OBE-SCL will allow me to be more flexible in using a variety of teaching methods in my class.	59 (49.2%)	34 (28.3%)	17 (14.2%)	10 (8.3%)
9.	I believe that the OBE-SCL approach will provide all of my students with equal educational opportunities.	55 (45.8%)	48 (40.0%)	11 (9.2%)	6 (5.0%)
	*Total =*	*46.04%*	*37.50 %*	*9.38%*	*7.08%*
	**Feelings**				
10.	I feel that OBE-SCL require more responsibilities from teachers/academics than content-driven (traditional) approach.	54 (45.0%)	47 (39.2%)	12 (10.0%)	7 (5.8%)
11.	I feel that OBE-SCL would not be a waste of time.	50 (41.7%)	57 (47.5%)	9 (7.5%)	4 (3.3%)
12.	I feel that traditional pen and paper tests to assess student competencies do not always benefit the students.	38 (31.7%)	57 (47.5%)	16 (13.3%)	9 (7.5%)
13.	I feel that OBE-SCL is the best learning approach.	48 (40.0%)	61 (50.8%)	7 (5.8%)	4 (3.3%)
14.	I feel that OBE-SCL will provide me with an opportunity to ensure that all learners achieve success.	52 (43.3%)	47 (39.2%)	14 (11.7%)	7 (5.8%)
	*Total =*	*40.33%*	*44.83%*	*9.67 %*	*5.17%*
	**Readiness**				
15.	I am willing to organize my daily schedule to have enough preparation time for OBE-SCL.	47 (39.2%)	50 (41.7%)	15 (12.5%)	8 (6.7%)
16.	I am willing to use any available resources to present my lesson using OBE-SCL.	56 (46.7%)	44 (36.7%)	8 (6.7%)	12 (10.0%)
17.	I believe that my experience in teaching will help me adapt to OBE-SCL in teaching and learning.	53 (44.2%)	33 (27.5%)	19 (15.8%)	15 (12.5%)
18.	I am willing to do a lot of subject-related readings in order to improve my knowledge and understanding of OBE-SCL.	52 (43.3%)	41 (34.2%)	17 (14.2%)	10 (8.3%)
19	I am willing to attend seminars and trainings relevant to the preparation and implementation of OBE-SCL at Afghan Public Universities.	44 (36.7%)	60 (50.0%)	9 (7.5%)	7 (5.8%)
	*Total =*	*42%*	*38%*	*11.33%*	*8.67%*
	**Acceptance Level**				
20.	I am willing to design the course outcomes and program outcomes aligned with the department/faculty/institutional outcomes.	60 (50.0%)	41 (34.2%)	11 (9.2%)	8 (6.7%)
21.	I am willing to deliver the written curriculum that has been designed in the course syllabi.	30 (25.0%)	50 (41.7%)	20 (16.7%)	20 (16.7%)
22.	I am willing to use different assessment methods and tools to evaluate students' progress.	57 (47.5%)	53 (44.2%)	10 (8.3%)	0 (0%)
23.	I am willing to assess students' progress using rubrics.	45 (37.5%)	41 (34.2%)	19 (15.8%)	15 (12.5%)
24.	I am willing to shift from the traditional approach (content-based) to OBE-SCL approach.	53 (44.2%)	58 (48.3%)	6 (5.0%)	3 (2.5%)
	*Total =*	*40.83%*	*40.50%*	*11%*	*7.67%*

Note: SA = Strongly Agree, A = Agree, D = Disagree, and SD = Strongly Disagree.

Totally, In terms of the knowledge level of all the respondents, Table 2 shows the level of their agreement and disagreement with the 5 items of the questionnaire asked from them. It shows that (14%) strongly agreed and (32.33%) agreed that they have the knowledge of how to use OBE-SCL in the classrooms and how to manage themselves with it. However, still, a visible number of respondents (34.17%) disagreed and (19.50%) strongly disagreed that they are still in the lower level of knowledge to utilize the approach in their classrooms. The higher disagreement is in terms of the equipment, classroom climate, the failure of providing cooperative and purposeful activities, and having the knowledge of using various types of assessment techniques to make sure that how the learning took place.

Moreover, most of the respondents believed the OBE-SCL approach to raise the academic performance of their students, requires more contacts with industry, having the flexibility of employing various types of teaching and learning methods, and providing equal opportunities to the students. It means that referring to Table 2., it seems that (46.04%) strongly agreed and (37.50%) of them agreed and believed in OBE-SCL as a good teaching and learning approach. However, (9.38%) are the respondents who still disagreed, and (7.08%) strongly disagreed with the items in that part.

In addition, approximately (40.33%) of the respondents strongly agreed and (44.83%) agreed and feel that OBE-SCL will require more responsibilities but it is not a waste of time, but it will make the students be active learners in the classrooms and when compared with the traditional (content-based) approach, it is the best learning approach and the traditional approach failed to benefit learners in this regard. The reason is that outcome-based education and student-centered learning focus on students, not the teachers compared with the traditional methods of teaching and learning. However, still (9.67%) disagreed and (5.17%) strongly disagreed with the items and preferred the traditional (content-based) approach of teaching and learning.

Furthermore, in terms of their readiness of whether or not the lecturers are ready to adapt to the new system, so almost (42%) of the respondents strongly agreed, and (38%) agreed that they are willing and will be ready for the implementation of OBE-SCL in their classrooms. It means that they are willing in terms of organizing the schedule and preparation, using the available resources, subject-related readings, attending seminars and workshops in regard to OBE-SCL and they are willing to adapt to the newly introduced teaching and learning approach by the MoHE and HEDP. However, still (11.33%) disagreed, and (8.67%) strongly disagreed that they are not ready to implement it in their classrooms.

Likewise, in terms of the acceptance of the new teaching and learning approach, a major number of the respondents (40.83%) strongly agreed, (40.50%) agreed and accepted that they have the willingness to formulate course outcomes, program outcomes to be aligned with institutional outcomes. Within, the willingness of delivering the written curriculum, using different assessing methods, and most importantly, shifting from the traditional (content-based) approach to the new (OBE-SCL) approach and using it in the higher education institutions. Nevertheless, a number of the participants (11%) disagreed and (7.67%) strongly disagreed in regard to the acceptance of OBE-SCL.

The following Table 3 outlines the descriptive statistics and percentages of the responses in regard to the implementation of OBE-SCL in the area of formulation of the learning outcomes.

**Table 3:** Implementing OBE-SCL in the area of formulating learning outcomes and their alignment.

Items	EH	MH	L	VL
1. I formulate the intended learning outcomes of the institution.	8 (6.7%)	13 (10.8%)	91 (75.8%)	8 (6.7%)
2. I formulate the program learning outcomes.	8 (6.7%)	11 (9.2%)	85 (70.8%)	16 (13.3%)
3. I formulate the course learning outcomes.	13 (10.8%)	29 (24.2%)	66 (55.0%)	12 (10.0%)
4. I formulate the students' learning outcomes as the instructional target.	18 (15.0%)	25 (20.8%)	67 (55.8%)	10 (8.3%)
5. I develop the learning outcomes for cognitive level domain.	21 (17.5%)	25 (20.8%)	62 (51.7%)	12 (10.0%)
6. I develop the learning outcomes for psycho-motor level domain.	30 (25.0%)	14 (11.7%)	68 (56.7%)	8 (6.7%)
7. I develop the learning outcomes in the affective level domain.	28 (23.3%)	17 (14.2%)	63 (52.5%)	12 (10.0%)
8. I construct the graduate outcomes primarily based on vision, mission statement(s) of the department/faculty/university.	17 (14.2%)	22 (18.3%)	68 (56.7%)	13 (10.8%)
*Total =*	*14.83%*	*16.25%*	*59.35%*	*9.57%*
**Alignment of Learning Outcomes**				
1. I align the program outcomes with the institutional outcomes.	11 (9.2%)	9 (7.5%)	85 (70.8%)	15 (12.5%)
2. I align the course learning outcomes with the program outcomes.	7 (5.8%)	15 (12.5%)	79 (65.8%)	19 (15.8%)
3. I align the instructional learning outcomes with the course learning outcomes.	10 (8.3%)	15 (12.5%)	80 (66.7%)	15 (12.5%)
4. I transform the course outcomes to long-term outcomes that are related to students' future life roles.	13 (10.8%)	8 (6.7%)	87 (72.5%)	12 (10.0%)
*Total =*	*8.57%*	*9.77%*	*68.93%*	*12.73%*

Note: EH = Extremely High, MH = Moderately High, L = Low, and VL = Very Low.

The results in Table 3 show that (14.83%) of the respondents formulate learning outcomes at an extremely high level and (16.25%) formulate moderately high. On the other hand, (59.35%) of the respondents still formulate them in a low and (9.57%) of them at a very low level.

Also, currently, the alignment of the outcomes (learning outcomes, course outcomes, and program outcomes) is in a low (68.93%) and very low (12.73%) level, and only (8.57%) of the teachers align them extremely high and moderately high (9.77%).

We can see in the following Table 4 which outlines the descriptive statistics and percentages of the responses in regard to the implementation of OBE-SCL in the area of curriculum content and structure.

**Table 4:** Implementing OBE-SCL in the area of curriculum content and structure.

Items	EH	MH	L	VL
1. I implement the learning plan as a guide to engage with the learners in the teaching-learning process.	11 (9.2%)	19 (15.8%)	68 (56.7%)	22 (18.3%)
2. I deliver the written curriculum that has been designed in the course syllabi.	17 (14.2%)	29 (24.2%)	52 (43.3%)	22 (18.3%)
3. I enhance the course syllabi that show the relationship of program outcomes with institutional outcomes and course outcomes to program outcomes.	11 (9.2%)	15 (12.5%)	68 (56.7%)	26 (21.7%)
4. I facilitate the students' learning to enhance knowledge and skills into a high-level performance.	17 (14.2%)	19 (15.8%)	73 (60.8%)	11 (9.2%)
5. I facilitate the curriculum contents to attain the learning outcomes.	14 (11.7%)	13 (10.8%)	81 (67.5%)	12 (10.0%)
*Total =*	*11.67%*	*15.83%*	*57%*	*15.50%*

Note: EH = Extremely High, MH = Moderately High, L = Low, and VL = Very Low.

The results of Table 4 show that as in the earlier parts of formulating outcomes in different levels (course/program), the results from the responses of the participants seem in the lower level of doing this. Also, the results show that (11.67%) of the respondents formulated extremely high, (15.83%) moderately high in terms of curriculum contents and structure. On the other hand, (57%) of them are still in the low and very low (15.50%) levels of implementing OBE-SCL in the area of curriculum content and structure.

Looking into the following Table 5., it seems that the descriptive statistics and percentages of the responses in regard to the implementation of OBE-SCL to the teaching and learning process in the Afghan context.

**Table 5:** Implementing OBE-SCL in the area of teaching-learning process.

Items	EH	MH	L	VL
1. I deliver instruction through student-centred (SCL) approach.	10 (8.3%)	30 (25.0%)	72 (60.0%)	8 (6.7%)
2. I align the teaching-learning activities and the intended learning outcomes.	13 (10.8%)	18 (15.0%)	82 (68.3%)	7 (5.8%)
3. I align the teaching-learning activities and the assessment task.	10 (8.3%)	14 (11.7%)	83 (69.2%)	13 (10.8%)
4. I align the teaching methods and strategies with the goals identified in the learning outcomes.	13 (10.8%)	17 (14.2%)	81 (67.5%)	9 (7.5%)
5. I identify the teaching and learning activities that facilitate the achievement of course learning outcomes.	16 (13.3%)	16 (13.3%)	78 (65.0%)	10 (8.3%)
6. I motivate the students' understanding of the outcomes they are meant to achieve.	12 (10.0%)	18 (15.0%)	79 (65.8%)	11 (9.2%)
7. I emphasize the knowledge and content (Cognitive domain) in the teaching and learning activities.	21 (17.5%)	30 (15.0%)	57 (47.5%)	12 (10.0%)
8. I emphasize students' skills and competencies (psycho-motor domain) in the teaching and learning activities.	11 (9.2%)	18 (15.0%)	66 (55.0%)	25 (20.8%)
9. I emphasize the values and attitudes (affective domain) in the teaching-learning activities.	14 (11.7%)	18 (15.0%)	66 (55.0%)	22 (18.3%)
10. I facilitate the learning activities for different types of learners in a diverse environment.	12 (10.0%)	19 (15.8%)	72 (60.0%)	17 (14.2%)
*Total =*	*11%*	*16.50%*	*61.33%*	*11.17%*

Note: EH = Extremely High, MH = Moderately High, L = Low, and VL = Very Low.

The teaching and learning process includes utilizing different teaching and learning activities and methods that are considered as one of the vital aspects of implementing OBE-SCL. As we can see in Table 5., the results show that implementing teaching-learning activities and methods in this process are neither extremely high (11%), nor moderately high (16.50%), but teachers use them in a low (61.33%) and a very low (11.17%) levels.

In addition, OBE-SCL utilizes various types of assessment methods in order to be able to align the process of teaching-learning. Therefore, the following Table 6 shows the descriptive statistics of the responses to what extent does the Afghan lecturers use different assessment techniques in order to ensure that they have achieved the learning outcomes and course outcomes.

**Table 6:** Implementing OBE-SCL in the area of assessing students’ knowledge, skills and attitudes.

Items	EH	MH	L	VL
1. I use different assessment tools to evaluate students' progress.	9 (7.5%)	13 (10.8%)	86 (71.7%)	12 (10.0%)
2. I assess students' knowledge.	16 (13.3%)	37 (30.8%)	59 (49.2%)	8 (6.7%)
3. I assess students' skills and competencies.	7 (5.8%)	12 (10.0%)	87 (72.5%)	14 (11.7%)
4. I assess students' values and attitudes.	9 (7.5%)	14 (11.7%)	84 (70.0%)	13 (10.8%)
5. I align the teaching methods and assessment.	11 (9.2%)	11 (9.2%)	71 (59.2%)	27 (22.5%)
6. I align the assessment procedure and tools with the learning outcomes.	12 (10.0%)	13 (10.8%)	85 (70.8%)	10 (8.3%)
7. I develop rubrics to assess the attainment of the institutional outcomes.	11 (9.2%)	16 (13.3%)	68 (20.8%)	25 (20.8%)
8. I develop rubrics to assess the attainment of program outcomes.	10 (8.3%)	11 (9.2%)	81 (67.5%)	18 (25.0%)
9. I develop rubrics to assess the attainment of course outcomes.	11 (9.2%)	13 (10.8%)	87 (72.5%)	9 (7.5%)
10. I assess the level of students' performance compared with the intended learning outcomes.	13 (10.8%)	14 (11.7%)	74 (61.7%)	19 (15.8%)
*Total =*	*9.03%*	*12.83%*	*65.17%*	*12.97%*

Note: EH = Extremely High, MH = Moderately High, L = Low, and VL = Very Low.

The results from Table 6 show that still, as in other parts of outcomes and teaching and learning process, teachers use the assessment methods in a low (65.17%) and very low (12.97%) levels in their teaching. However, it seems that a limited number of respondents use it in an extremely high (9.03%) and moderately high (12.83%) levels in their classrooms.

**Figure 1:**
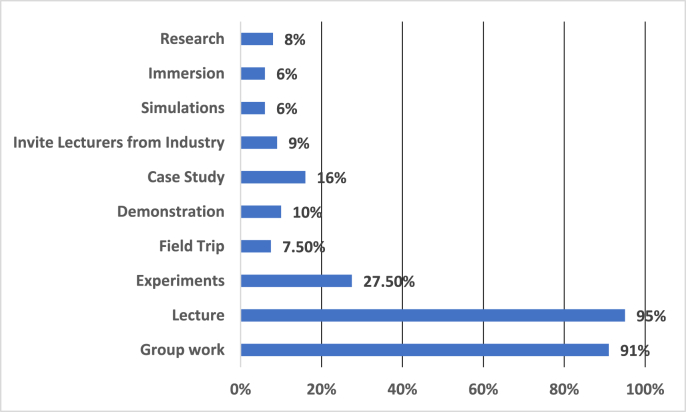
Teaching and learning strategies that lecturers use in classrooms.

Using student-centered strategies is one of the vital aspects of outcome-based education. For this reason, the success of OBE-SCL is highly dependent on the active and appropriate use of the teaching and learning strategies inside and outside of the classrooms. As it can be seen in Figure 1 bellow, Afghan teachers use group work (91%), lectures (95%), experiments (27.50%), field trips (7.50%), demonstration (10%), case study (16%), inviting lecturers from industry (9%), simulations (6%), immersion (6%) and research (8%) in different level of percentages.

**Figure 2:**
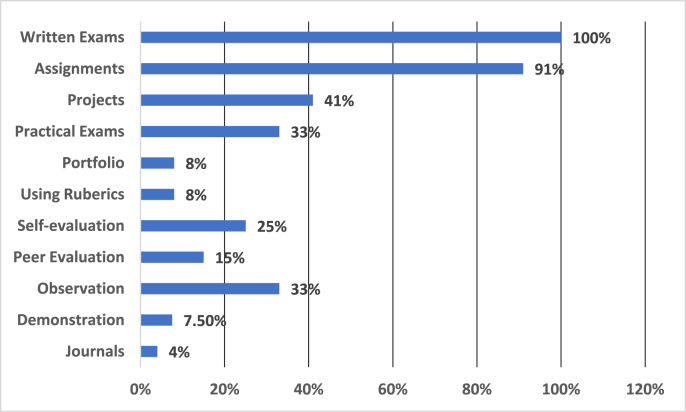
Assessment methods that lecturers use for grading students.

As Figure 2 indicate the level of the assessment methods used by lecturers currently, it seems that the tendency is still towards the traditional methods of assessment, written exams, and all of the respondents (100%) selected that they use it as an assessment tool for assessing the cognitive, affective and psychomotor levels of their students. However, using assignments (91%) as an assessment method is also considered vital by the teachers. Furthermore, using projects (41%), practical exams (33%), portfolios (8%), rubrics (8%), self-evaluation (25%), peer-evaluation (15%), observation (33%), demonstration (7.50%) and journals (4%) are also in practice by the Afghan lecturers, but when compared, it is in a low level, and in some types in a very low level.

### 4.2 Qualitative results

In this section, the qualitative results will be presented. Therefore, the following Table 7 highlights the qualitative responses of the seven participants that the researcher interviewed. The interviews have been analyzed based on the (nodes) using NVIVO 12.

**Table 7:** Interview responses of the participants.

Themes	Participant 1	Participant 2	Participant 3	Participant 4	Participant 5	Participant 6	Participant 7
What is OBE-SCL?	OBE is the planning… SCL is the soul for this planning… Without soul, you cannot do anything.	OBE…to identify what graduates be in the future? SCL helps to achieve this goal.	OBE is a philosophy focusing on outcomes… SCL is an approach…	OBE is an educational model rejects the traditional ones…	OBE… clear picture of what your graduates will be… SCL is an instructional approach…	OBE… a clear picture in terms of assessment, curriculum, teaching and learning …. SCL is a learning model.	OBE is an educational system focusing on outcomes… SCL is an approach focusing on students…
Developing the POs of Institutions	Not yet. We are working on it to develop.	No. but we are close to make them finalized.	No. we do not have POs for our institution yet…	Yes… but not developed based on OBE…	…working on it, soon will define our PEOs, COs, Pos…	Our faculty does not have PO until now	No. we have plan to make it.
Aligning POs, COs, and LOs	This is very important. Each of this is you know like a brick, a brick have to come up with the house. Without this planning, you can not make a house…	Very important. Otherwise, you know, its, OBE is about alignment of all this. It will not work if you do not align them all together…	…very closely related. ..if you want to ensure that the students at the end of the program, of the four years, they are supposed to have…	Outcome alignment is very important in OBE-SCL. Outcomes in all levels should match with one another.	Very important; should support each other and the vision and mission of the faculty/institution. These learning outcomes should explain our graduates…	For OBE and SCL is very important the alignment of outcomes because without the alignment of outcomes it is impossible to achieve OBE and SCL goals.	While practicing OBE system alignment is very important because COs, POs, and LOs must be aligned…
Students' Assessment Marks	Based on the regulations of MoHE, I assess students with 10% of assignments and classroom activities, and 80% of marks are exam oriented.	Usually, 10 marks for assignment. The other marks are given based on the mid-term and final exams.	I give 10 marks for assignments. The remaining marks for their paper based exams and 10 marks of attendance.	Out of 100 marks, I give 40 marks for the different assignment. 60 other marks are the midterm and final exam.	Currently we follow the rules of MoHE. Important to be mentioned, we work to finalize a sample of course policy based on OBE.	we still give marks to students' assignment according to traditional system (10 marks).	10 marks carries for attendance and class participation, 10 marks for project and practical works and 20 marks for internal exam and 60 marks for final exam…
Paradigm Shift of Traditional System	…beginning is quite difficult to change. So, the first thing to be aware is to give an awareness to our lecturers.	In program level, to ensure the curriculum is properly designed, collaboration among faculty...	I feel hopeful to this. But it needs to happen gradually.	If the system is fully upgraded to OBE-SCL, will be a great achievement in the education sector…	It will be difficult to fully upgrade at one instance the whole system, it will help gradually, and the MoHE is working on it…	…Difficult to fully upgrade OBE-SCL now; we still have a lot of challenges as our higher education ministry until do not have any POs…	I think it might take time to implement fully upgraded OBE system…
Readiness for OBE-SCL Model	Yes, I am ready. But it needs to be applied gradually. First, the workload of the lecturers must be decreased, content-based regulations should be changed...	Currently, yes, I am interested to implement OBE-SCL in my classroom. But sometimes the facilities will create problems…	Yes I am ready, but in case of removing all the obstacles towards it will give me the opportunity to implement it well.	Considering the current educational level, I am ready to implement OBE-SCL in my classrooms in case I have fewer classes: up to three or four classes.	Yes, already started the implementation, we are in the introduction phase, a country wise OBE-SCL committee is structured, have regular meetings and start the practical implementation of OBE-SCL.	I am ready but it is difficult to implement OBE_SCL because until now all of our higher education policies according to traditional system such as we do not have POs…	OBE is not related only to teachers, it needs many efforts at top management level, we do not have POs and neither we have PEOs at department and faculty levels. Currently I practice only COs and SCL…
Challenges towards the Change to OBE-SCL Model	Workload and administrative activities prevent organizing good classroom tasks, good assessment methods and so on. I teach 6–8 subjects in a single semester, hard for me applying initiatives…	I think large class size, not enough equipments and facilities and even not enough infrastructure…	Info-structure, infrastructure, mindset of the teachers, and overload are among the key obstacles…	…major obstacle is overload of teaching. I usually teach between six to 9 courses in every semester.	…low awareness, curriculum incompatibility, rules, and regulations…	traditional methods and policies, lack of lecturers' knowledge regarding OBE and SCL, lack of students' communicational and technological skills. Firm curriculum…	A large number of courses I teach, less qualified students introduced through Kankor, less equipment for teaching, unsuitable classrooms, limit access to internet for both teachers and students.
Other Challenges	The philosophy behind OBE must be understood... means sometimes teachers does not know the philosophy…	The lecturers' understanding and the university management…	The mindset, freedom for the lecturers and as well as autonomy of institutions…	unequipped classroom, stable electricity, labs, LCD, Projectors and so on.	The low awareness or improper mind-set…	Lack of internet, electricity, lecturers' large classes, students' technological, communication skills, labs	Large number of students introduced through Kankor and our curriculum never considered for the enrolment…

Table 7 presents the qualitative findings of the current study. As explained earlier, the researcher interviewed seven OBE-SCL trainers and master trainers in terms of the implementation of this approach at the Afghan public universities. The first item provided asks for the general definition of the approach. The raw data show that most of the respondents defined OBE-SCL as its common definition of focusing on outcomes and student-centered learning in educational institutions. P1 stated that OBE is the planning stage, and SCL is like the soul for this planning stage, where without incorporating it, outcome-based education can not take place. P3 viewed OBE as a philosophy, which focuses on the outcomes, and SCL is an approach.

Furthermore, in terms of the outcomes of the institutions in Afghanistan, all of the participants mentioned that they have not yet developed the program outcomes for their institutions. Only P4 stated that his institution had developed the program outcomes, but these outcomes are not based on the criteria of OBE.

In addition, when the participants were asked about the significance of the alignment of the program outcomes, course outcomes, and learning outcomes with each other, so they mentioned that aligning them all is very much important that helps institutions to achieve their outcomes. P2 mentioned that actually, OBE is about the alignment of all these, and without linking them closely together, OBE will not work as an effective approach.

Likewise, in terms of the assessment marks for the students, almost all of the participants claimed that they still assess students based on the traditional assessment procedures. They state that they follow the rules of the Ministry of Higher Education of Afghanistan, where the assessment is exam-oriented, not task-oriented. Therefore, almost all of them mentioned that they usually give students 10 marks for their assignments, and the remaining 90 marks are for the attendance (10 marks) and mid-term (20 marks), and final (60 marks) exams. Only P4 stated that he gives 40 marks for the assignments of the students, and the remaining 60 marks are given to them in the mid-term and final paper-based exams.

While the participants were asked about the paradigm shift from the traditional content-based educational system to the modern outcome-based system, they mentioned their willingness for this change by mentioning some concerns in this regard. Therefore, they mentioned that the change would only be possible to happen gradually, and they hoped that this would be a great achievement if the old system changes. For these reasons, they showed their readiness for this type of change with some prior steps to be taken before the change to come. For example, the main concern to be highlighted before the change is to make sure that the overload of the lecturers is the first factor that influences their readiness for the adaptation. Therefore, they mentioned that this seriously affects their preparation for teaching and avoids proper planning of the outcome-based and student-centered activities.

In terms of the obstacles that influence the process, participants mentioned numerous challenges that avoid the proper implementation of the approach. The most obvious reasons that they emphasized were; the workload of their subjects, large class size, lack of equipment and facilities, lack of enough info-structure and infra-structure, the mindset of the teachers and faculty members, low awareness, curriculum incompatibility, traditional rules, and regulations are among the challenges that most of them focused on. In addition, the lack of students’ communication and technological skills is also considered (P6) a problem in this process. It means that as student-centered learning is the core of outcome-based education, so most of the students are not good communicators and unfamiliar with how to communicate and how to use technology, which are very important factors of student-centered learning. The other challenges that the participants mentioned were the difficulty of understanding the philosophy of OBE, the poor management of the institutions, and no financial autonomy of institutions are among the other key ones they mentioned.

## 5 Discussion of the results

The results of the current study have been discussed based on the research questions of the current study in the following sections.

### 5.1 What are the attitudes of Afghan lecturers towards OBE-SCL?

Based on the results obtained for the current study, the attitudes of lecturers seem positive towards OBE-SCL. As presented in Table 2 above, it seems that the attitudes of the Afghan lecturers in terms of beliefs, feelings, readiness, and acceptance level are quite positive towards the implementation of OBE-SCL. This is supported by the studies conducted by (Alimyar, 2020; Baguio, 2019) and the findings of their studies reveal that the participants had very favorable and positive attitudes towards the implementation of OBE. However, in terms of their knowledge level, the results of the current study show that almost half of the respondents still not fully understand this approach. It might be because most of them (As in Table 1.) have not yet participated in any training workshops related to OBE-SCL inside or outside of their institutions. Therefore, more workshops and training need to be organized in order for the teachers to understand the philosophy of OBE-SCL as this is also supported by the findings of Alimyar (2020), who explored the efficacy of the training workshops for the Afghan lecturers. His research findings outlined that the lecturers who participated in OBE-SCL-related workshops were positively impacted and implemented this approach in their classrooms. In terms of their beliefs and feelings as presented in Table 2., it seems that the participants felt and believed OBE-SCL as an effective and flexible approach for teaching and learning compared with the traditional content-based approach. Similarly, most of the respondes showed thier readiness for implementing OBE-SCL and accepted this approach that they are willing to shift from the traditional content-based teaching and learning approach to this modern one. As in the study of Ortega and Cruz (2016), the attitudes of the participants were quite positive towards the OBE approach of teaching and learning. However, despite their willingness and readiness for the paradigm shift, without organizing enough training workshops, the practical implementation of the outcome-based education will not be that much useful as would be.

Based on the qualitative results, as the participants of this part joined multiple times in the training regarding this approach, so their knowledge and attitudes were very positive about it. It means that they showed their willingness and readiness that they have enough knowledge about OBE-SCL related matters and have the ability to implement it in their classrooms. However, the interview scripts also indicate that in terms of shifting from the traditional system to the new one, they emphasized that it should take place gradually. They focused on the mindset first for students, teachers, and administrative staff in order to be mentally prepared for the change.

As Jansen (1998) also stated that the successful implementation of OBE-SCL would require enough training workshops for the faculty members to be able to understand the philosophy, assessment procedures and to be able to have the ability of continuous monitoring. Mogashoa (2013) also mentioned that lack of enough training for teachers will lead this process to its failure. Therefore, despite the positive attitudes that the participants showed towards OBE-SCL, it is still needed for them to have trained with enough knowledge and understanding in terms of not only the theoretical framework of the OBE-SCL but in terms of practical implementation measures as well to make sure that the theory and practice are considered and applied equally.

### 5.2 What is the extent of implementing OBE-SCL at Afghan public universities?

Based on both qualitative and quantitative results obtained in this study, it seems that implementing the approach is in its early phase of implementation. For example, the formulation of the learning outcomes is still at a lower level in most of the institutions. The quantitative data show that the formulation of outcomes (Program, Course, and Learning Outcomes) are still at a low level and most of them did not even develop them at all (See Table 3.). Similarly, in terms of the alignment of learning outcomes to the program and course outcomes, its rate is also in the lower level of implementation. Unlike the research of Baguio (2019), which shows that the respondents implementing OBE in extremely high level. His study also shows that the main focus of the respondents was on the alignment of all these outcomes. As the interview scripts of the current study also support this idea that most of the respondents claimed that their institutions still have not developed program outcomes yet. It means that the number of teachers who developed learning outcomes might be the ones who participated 2–5 times in the OBE-SCL related training as well those who are holding higher degrees of master and Ph.D. would have developed them. In a research study, Tshai et al. (2014) focused on two main elements of OBE-SCL and as they stated, they are PEOs and as well as POs which they considered as of much importance for this model. As the participants mentioned understanding the philosophy of OBE-SCL is one of the important elements of implementing this approach, so the lack of enough knowledge will negatively affect this process. Mogashoa (2013) also pointed out that teachers should understand the philosophy of the model to be able to align the outcomes and as well as the assessment procedures to ensure the successful implementation of OBE-SCL.

In terms of the implementation in the area of curriculum content and structure, it seems that it is also in a low level of implementation. As shown in Table 4, a small number of respondents implemented it in extremely high and moderately high levels. Also, the qualitative findings show that the curriculum structure and learning contents (textbooks) are still based on the traditional principles as the study of Katawazai et al. (2019) also investigated the low quality of the textbooks, and this causes OBE-SCL to be implemented in a small extent in this area. So, it means that the current curriculum and the contents are not able to ensure the attainment of the outcomes. Unlike the findings of the research study conducted by Baguio (2019) which shows that the majority of the participants responded as they implement curriculum content and structure as extremely high and the main focus of them was on the curriculum content of attaining the learning outcomes.

Similarly, in terms of using different kinds of activities in the area of teaching and learning process, quantitative findings reveal that it is also in a low level of implementation as in Table 5. It means that teachers still rely on the teacher-centered methods rather than the student-centered method as the study of Ahmadzai et al. (2019) also found it. It is unlike the findings of Baguio (2019) who stated that the respondents use different types of activities in the area of teaching and learning and the main focus is on the values and attitudes to be emphasized during these activities. Also, the recent study conducted by Katawazai and Saidalvi (2020) about the attitudes of Afghan students towards cooperative learning strategies at the tertiary level, shows that the attitudes of students are quite positive towards using cooperative learning strategies and implementing such strategies increased the active participation in the classroom activities.

Likewise, the assessment process is also being implemented based on traditional principles, not modern ones. The majority of the respondents still use the traditional procedures of the assessment and a small number of them use the modern methods of assessment. Therefore, as Table 6 also presents, it seems that using different assessment tools to assess students’ knowledge, skills, competencies, values, and attitudes is still at a low level in this process. As well as regarding the assessment methods and teaching and learning strategies (Figures 1 and 2), it seems that most of the teachers still use written exams (100%) as a dominant assessment procedure. Using assignments (91%) is in the second place that teachers use. The remaining methods of assessment were rarely used by teachers. Also, the dominant teaching and learning strategies were the lecturing method and group work. This is similar to the findings of Loreto (2018) where the focus of the instructors is also on three methods such as; lecture, group work, and case study. Also, instructors heavily relied on exams in terms of assessment procedures.

The qualitative findings of the current study also show that teachers still use exam-oriented assessment procedures and they assess students’ abilities and skills based on written exams (60–80%). The reasons that the participants mentioned in this regard were that they still follow the rules and regulations of the Ministry of Higher Education of Afghanistan where only 10% of the marks are given to the assignments, the remaining marks are for the attendance, mid-term and final paper-based examinations. Thus, the extent of implementing OBE-SCL is still at its low level in terms of formulating outcomes, curriculum, teaching and learning strategies, and assessment methods.

### 5.3 What are the challenges of implementing OBE-SCL in the Afghan context?

As the results about the practice of the OBE-SCL model show that it is still in the lower level of implementation, so the participants of the qualitative part also mentioned some of the challenges that they think avoid the implementation of this approach in the Afghan context.

The first challenge that they mentioned is in terms of the content-based rules and policies that MoHE still emphasizes institutions to apply them for teaching and learning. For example, in the assessment procedure, teachers should give (60–80%) marks to the mid-term and final exams, and the classroom activities and assignments carry only (10%) of the marks. The remaining (10%) of the marks are given to the attendance of the students. In a research study, Jansen (1998) investigated the challenges of the model in the South African context and stated that the assessment policy is problematic if the teachers are not fully trained for that to implement in the classrooms.

Within, the content-based curriculum is designed based on the traditional procedures. Teachers emphasized that all the statements, goals, and objects should be developed in OBE-SCL and modern teaching and learning activities should be placed in the curriculum. In addition, teachers stated that their workload avoids their readiness to plan and utilize OBE-SCL activities in their classrooms. They claimed that even sometimes they teach (6–9) subjects in a single semester and they do not have enough time to organize their workload with the implementation of OBE-SCL. They mentioned that in this case, they only have a very short time to share the contents of textbooks with students without using some innovative ways or learning activities to be used in the classroom. Jansen (1998) stated that teachers should be given enough time to be able to manage the classroom tasks based on the OBE-SCL model appropriately.

Within the workload, they complained about the limited or no facilities in their classrooms. For example, they stated that even they do not have access to a medium speed internet, and as well as there is no electricity in the institutions, particularly those located in the provinces. Also, there is no projector in most of the classrooms, not enough and appropriate chairs and tables in the classroom to assign students to work in cooperative groups. Mogashoa (2013) focused on having adequate resources that will lead the model to its successful status. The researcher believed that the lack of enough resources would be counted as a major obstacle towards its success.

In addition, the lack of infrastructure (enough classrooms) and info-structure (internet facilities) and also large class size are the other key challenges that must be considered seriously. Jansen (1998) also considered large classroom size as one of the problematic obstacles towards implementing the OBE-SCL model in the classrooms. Similarly, Ortega and Cruz (2016) also stated that the respondents of their study pointed out the large classroom size as one of the major constraints that negatively influence the teaching and learning process and it is considered as an obstacle towards the OBE approach.

As well, most of them mentioned that their institutions still have not developed POs, and they are unable to develop their course and learning outcomes based on the POs of the institution and to include them in the course policies for aligning the teaching and learning activities and learning outcomes with the assessment procedures. As Rao (2020) also discussed the significance of attaining learning outcomes, aligning them with the program and course outcomes, and as well as associating them with the three domains of Bloom's Taxonomy. Due to the challenges mentioned by the participants of this study, it is difficult for the lecturers to be able to implement the OBE-SCL approach in their classrooms properly. Therefore, much more practical and measurable steps are needed to be taken in resolving these challenges and facilitating the implementation of this model in higher education institutions. For this reason, the adaptation from the traditional (content-based) system to the OBE-SCL system will impact the quality of education and will help institutions to produce capable graduates based on the needs of the market and society.

## 6 Conclusions

The current study aimed to investigate the attitudes of the lecturers regarding the implementation of OBE-SCL at Afghan public universities, and the extent of the practices and challenges. So, exploring the attitudes of the teachers, the extent of the implementation, and the challenges will draw an overall picture of the current level of OBE-SCL in the Afghan context. The attitudes of teachers are encouraging, and they tend to implement this approach in their teaching and learning process. Teachers who responded positively were the ones who at least participated in the training related to this approach and they were optimistic about the implementation process of this model. However, still, a visible number of the participants responded negatively about the implementation of the approach at the tertiary level of the Afghan context. It might be because there still seem (34.2%) of the respondents had even not participated in any type of training workshop related to OBE-SCL inside and outside of their institutions. For this reason, most of the respondents claimed their readiness for the implementation of OBE-SCL in their classroom, but they have also indicated some obstacles and proposed some suggestions to be considered as pre-requisite factors prior to the implementation of OBE-SCL. As for the extent of the approach, it would seem that the implementation process is at its low level and much more efforts are required to track the process and remove the barriers that prevent the approach from being fully implemented.

In terms of making the attitudes of the teachers more positive in the Afghan context, intensive training workshops should be structured by the strong commitment of institutions and the PDCs (Program Development Centers) and other centers in order to share the big picture of OBE-SCL and its professional development with all the faculty members. These kinds of training need to be carried out by experienced individuals, particularly, trainers and master trainers of OBE-SCL in order to share numerous ways of implementing the approach thoroughly.

As Gurukkal (2020) also mentioned the potential of OBE in the global bigger context, so it is mandatory for the higher education institutions to adapt with the outcome-based educations system because of the ongoing economic revolution that the global growth of market needs such kinds of standardized indicators and international accreditations measures that higher educations need to implement.

## 7 Recommendations

Although it is obvious that OBE-SCL is in its early stages in the Afghan context, it needs much more focus and commitment at the governmental and organizational level when moving to its further implementation phases. For the effective implementation of the OBE-SCL, the participants of this study suggested the following recommendations to be considered in the Afghan context in the policymaking process for this model:

1. Implementation of OBE-SCL to be practiced even at the school level, particularly, in secondary education because students join universities with low technological and communicational skills which are the requirements for lecturers implementing OBE-SCL-related activities in the tertiary level classrooms. This causes that for the first time, students experiencing OBE-SCL methods at university for which they are not mentally ready. Thus, before enrolling in tertiary education, students should have some basic communication skills, group working skills, basic technological skills (at least computer and internet skills), and a basic manner for being ready for SCL while working in curricular and extra-curricular activities.
2. To better implement OBE-SCL in Afghanistan, the Ministry of Higher Education should first determine the teaching obligations and responsibilities of the teaching staff rightly that how many classes should each teaching staff teach and ask the universities to announce teaching vacancies if they require to decrease the teaching load of the lecturers. Within, it should send an official letter to all the public universities to implement OBE-SCL without any excuses and to emphasize it. Then, MoHE should have a policy of reward and punishment. It means that it has to be strictly followed by all whether the universities implement OBE-SCL or not, and to reward all those lecturers who are practically implementing OBE-SCL in their classrooms.
3. A very best effort of the management and a strong commitment of the administrative staff is required to make sure that the vision and mission statements clearly covers the ethical, social, and economical values of Afghanistan to be in link with the market and the needs of the stakeholders and to have a national committee/board or some executive agency should regularly follow the development of OBE-SCL implementation in the country and as well as in institutions.
4. MoHE and institutions should be able to provide some basic facilities in order to be able to use them while implementing OBE-SCL such as internet access for both teachers and students, electricity (in order to be able to use the available resources in the classrooms), projectors in the classrooms, chairs, and tables appropriate for different group working activities, basic equipment for labs in different disciplines.
5. Changing MoHE content-based regulations regarding assessment, examinations, assignments, and the roles of teachers to be facilitators in classrooms, not the authority, as well as the roles of the students to be active participators during all the classroom activities, not only the passive receivers of information.
6. Within, the workload of the teachers that most of the time each teacher teaches (4–8) subjects is an obstacle towards the readiness and implementation of OBE-SCL and they do not have enough time for preparation and initiative activities.
7. Allocating enough money for revising the current content-based curriculum. It means that teaching and learning activities are still based on traditional methods, so all of them should be re-designed based on OBE-SCL. As well, the PEOs, POs, and COs need to be structured and aligned well which should be in relation to all the statements of the institution and as well as with the needs and feedback of the stakeholders and market.
8. Increasing the number of OBE-SCL training workshops inside and outside the institution for the faculty members. As we have seen in the results as well that a visible number of the participants in the current study were not even participated in such kinds of training workshops and due to this deficiency, they failed to implement OBE-SCL in their classrooms or to at least know that what it is about.
9. As institutions still have not designed their POs and it avoids designing the course and learning outcomes, so MoHE should give some freedom to the public institutions in terms of developing program outcomes in order to be able to start the implementation of the OBE-SCL properly. Within, institutions should be given financial autonomy as well to be able to prepare training, to purchase equipment for teachers and classrooms, and to be able to have a planning for the workshops and facilities that they need for their academic, teaching, and learning activities.

## 8 Limitations of the research

1. The sampling size for the current study is not enough and the findings and results of the current study would not be able to be generalized due to this small number of participants.
2. Rejecting to participate in the study caused a limited number of participants to take part in the current study because they stated that they do not know about the OBE-SCL at all and were not willing to take part in this research study.
3. The respondents were from a limited number of public universities and the lecturers of some public universities did not participate at all. Due to this limitation, the results would not be applicable to all public universities.
4. The current study only covers the attitudes of teachers regarding OBE-SCL, not the students. For this reason, it needs further research studies to be conducted in order to include both students and teachers in a study to have them both as participants.

## Declarations

### Author contribution statement

Rahmatullah Katawazai: Conceived and designed the experiments; Performed the experiments; Analyzed and interpreted the data; Contributed reagents, materials, analysis tools or data; Wrote the paper.

### Funding statement

This work was supported by the Ministry of Higher Education and Higher Education Development Program, Afghanistan.

### Data availability statement

Data will be made available on request.

### Declaration of interests statement

The authors declare no conflict of interest.

### Additional information

No additional information is available for this paper.

## Appendix A Supplementary data

APPENDIX 1 The Questionnaire.docx APPENDIX 1 The Questionnaire.docx
