# Supplementary material for: Implementing outcome-based education and student-centered learning in Afghan public universities: the current practices and challenges
Source: Heliyon. 2021 May 21;7(5):e07076. doi: 10.1016/j.heliyon.2021.e07076 (PMC8165413; doi:10.1016/j.heliyon.2021.e07076)
Supplement: APPENDIX 1 The Questionnaire.docx [file mmc1.docx]

**APPENDIX 1**

**QUESTIONNAIRE**

Implementing Outcome-based Education and Student-centered Learning in Afghan Public Universities: the Current Practices and Challenges

Dear Participant!

As you know that the Ministry of Higher Education of Afghanistan and HEDP intended to change the traditional (content-based) educational system with the modern (Outcome-based education & Student-centred learning) at public universities of our country. The aim of the current study is to investigate the attitudes of university lecturers towards the implementation of OBE-SCL in the Afghan context and to identify the current practices of this model into classrooms and the challenges that Afghan lecturers are being faced with while utilizing OBE-SCL in their institutions/departments/classrooms. The following Questionnaire asks for your opinion on a number of issues relating to (OBE-SCL) in the Afghan context. By completing this Questionnaire, you can help the researcher gain an understanding of your attitudes, perceptions, and feelings towards OBE-SCL and its challenges in the Afghan context. Your answers will also be helpful indicators of the challenges that what is still needed to successfully implement OBE-SCL in the Afghan context. I would like to assure you that the response will be used only for the purpose of the current research and will keep confidential.

The success of the current study is highly depending on the accurate and relevant information that you will provide. So, you are kindly requested to read each question carefully and to give your genuine response to each item of the questionnaire honestly.

Thank you very much for your assistance in this regard!

Are you ready to participate in this study? Yes No

***Section I: Demographic Information***

1. Gender □ Male □ Female
2. Age □ 25-30 □ 31-35 □ 36-40 □ Above 40
3. Teaching Experience □ 1-5 Years □ 6-10 Years □ 11-15 Years □ Above 25 Years
4. Educational Degree □ Bachelor □ Master □ PhD
5. Name of the University you teach ( )
6. How many times have you participated in OBE-SCL workshops/seminars inside or outside the university? □ 1 Time □ 2 Times □ 3-5 Times □ Above 5 Times □ Not Participated Yet

***Section II The Attitudes of Lecturers***

**A. Knowledge Level**

1. I know where to start OBE-SCL in my class.

□ **Strongly Agree** □ **Agree** □ **Disagree** □ **Strongly Disagree**

1. I know how to facilitate an outcomes-based class.

□ **Strongly Agree** □ **Agree** □ **Disagree** □ **Strongly Disagree**

1. I am able to align the world of teaching with the world of working.

□ **Strongly Agree** □ **Agree** □ **Disagree** □ **Strongly Disagree**

1. I am equipped to make OBE-SCL classroom climate, providing cooperative, well-directed and purposeful activities.

□ **Strongly Agree** □ **Agree** □ **Disagree** □ **Strongly Disagree**

1. I have enough knowledge of the assessment techniques in OBE-SCL.

□ **Strongly Agree** □ **Agree** □ **Disagree** □ **Strongly Disagree**

**B. Beliefs**

1. I believe that OBE-SCL will improve students’ academic achievements.

□ **Strongly Agree** □ **Agree** □ **Disagree** □ **Strongly Disagree**

1. I believe that OBE-SCL would require more contacts and communication with industry.

□ **Strongly Agree** □ **Agree** □ **Disagree** □ **Strongly Disagree**

1. I believe that OBE-SCL will allow me to be more flexible in using a variety of teaching methods in my class.

□ **Strongly Agree** □ **Agree** □ **Disagree** □ **Strongly Disagree**

1. I believe that the OBE-SCL approach will provide all of my students with equal educational opportunities.

□ **Strongly Agree** □ **Agree** □ **Disagree** □ **Strongly Disagree**

**C. Feelings**

1. I feel that OBE-SCL require more responsibilities from teachers/academics than content-driven (traditional) approach.

□ **Strongly Agree** □ **Agree** □ **Disagree** □ **Strongly Disagree**

1. I feel that OBE-SCL would not be a waste of time.

□ **Strongly Agree** □ **Agree** □ **Disagree** □ **Strongly Disagree**

1. I feel that traditional pen and paper tests to assess student competencies do not always benefit the students.

□ **Strongly Agree** □ **Agree** □ **Disagree** □ **Strongly Disagree**

1. I feel that OBE-SCL is the best learning approach.

□ **Strongly Agree** □ **Agree** □ **Disagree** □ **Strongly Disagree**

1. I feel that OBE-SCL will provide me with an opportunity to ensure that all learners achieve success.

□ **Strongly Agree** □ **Agree** □ **Disagree** □ **Strongly Disagree**

**D. Readiness**

1. I am willing to organize my daily schedule to have enough preparation time for OBE-SCL.

□ **Strongly Agree** □ **Agree** □ **Disagree** □ **Strongly Disagree**

1. I am willing to use any available resources to present my lesson using OBE-SCL.

□ **Strongly Agree** □ **Agree** □ **Disagree** □ **Strongly Disagree**

1. I believe that my experience in teaching will help me adapt to OBE-SCL in teaching and learning.

□ **Strongly Agree** □ **Agree** □ **Disagree** □ **Strongly Disagree**

1. I am willing to do a lot of subject-related readings in order to improve my knowledge and understanding of OBE-SCL.

□ **Strongly Agree** □ **Agree** □ **Disagree** □ **Strongly Disagree**

1. I am willing to attend seminars and trainings relevant to the preparation and implementation of OBE-SCL at Afghan Public Universities.

□ **Strongly Agree** □ **Agree** □ **Disagree** □ **Strongly Disagree**

**E. Acceptance Level**

1. I am willing to design the course outcomes and program outcomes aligned with the department/faculty/institutional outcomes.

□ **Strongly Agree** □ **Agree** □ **Disagree** □ **Strongly Disagree**

1. I am willing to deliver the written curriculum that has been designed in the course syllabi.

□ **Strongly Agree** □ **Agree** □ **Disagree** □ **Strongly Disagree**

1. I am willing to use different assessment methods and tools to evaluate students’ progress.

□ **Strongly Agree** □ **Agree** □ **Disagree** □ **Strongly Disagree**

1. I am willing to assess students’ progress using rubrics.

□ **Strongly Agree** □ **Agree** □ **Disagree** □ **Strongly Disagree**

1. I am willing to shift from the traditional approach (content-based) to OBE-SCL approach.

□ **Strongly Agree** □ **Agree** □ **Disagree** □ **Strongly Disagree**

*Section III OBE Implementation in the Area of Formulation and Alignment of Learning Outcomes*

**A. Formulation of Learning Outcomes**

1. I formulate the intended learning outcomes of the institution.

□ **Extremely High** □ **Moderately High** □ **Low** □ **Very Low**

1. I formulate the program learning outcomes.

□ **Extremely High** □ **Moderately High** □ **Low** □ **Very Low**

1. I formulate the course learning outcomes.

□ **Extremely High** □ **Moderately High** □ **Low** □ **Very Low**

1. I formulate the students’ learning outcomes as the instructional target.

□ **Extremely High** □ **Moderately High** □ **Low** □ **Very Low**

1. I develop the learning outcomes for cognitive level domain.

□ **Extremely High** □ **Moderately High** □ **Low** □ **Very Low**

1. I develop the learning outcomes for psycho-motor level domain.

□ **Extremely High** □ **Moderately High** □ **Low** □ **Very Low**

1. I develop the learning outcomes in the affective level domain.

□ **Extremely High** □ **Moderately High** □ **Low** □ **Very Low**

1. I construct the graduate outcomes primarily based on vision, mission statement(s) of the department/faculty/university.

□ **Extremely High** □ **Moderately High** □ **Low** □ **Very Low**

**B. Alignment of Learning Outcomes**

1. I align the program outcomes with the institutional outcomes.

□ **Extremely High** □ **Moderately High** □ **Low** □ **Very Low**

1. I align the course learning outcomes with the program outcomes.

□ **Extremely High** □ **Moderately High** □ **Low** □ **Very Low**

1. I align the instructional learning outcomes with the course learning outcomes.

□ **Extremely High** □ **Moderately High** □ **Low** □ **Very Low**

1. I transform the course outcomes to long-term outcomes that are related to students’ future life roles.

□ **Extremely High** □ **Moderately High** □ **Low** □ **Very Low**

*Section IV OBE Implementation in the Area of Curriculum Content and Structure*

**A. Curriculum Content and Structure**

1. I implement the learning plan as a guide to engage with the learners in the teaching-learning process.

□ **Extremely High** □ **Moderately High** □ **Low** □ **Very Low**

1. I deliver the written curriculum that has been designed in the course syllabi.

□ **Extremely High** □ **Moderately High** □ **Low** □ **Very Low**

1. I enhance the course syllabi that show the relationship of program outcomes with institutional outcomes and course outcomes to program outcomes.

□ **Extremely High** □ **Moderately High** □ **Low** □ **Very Low**

1. I facilitate the students’ learning to enhance knowledge and skills into a high-level performance.

□ **Extremely High** □ **Moderately High** □ **Low** □ **Very Low**

1. I facilitate the curriculum contents to attain the learning outcomes.

□ **Extremely High** □ **Moderately High** □ **Low** □ **Very Low**

*Section V OBE Implementation in the Area of Teaching-Learning Process*

1. **Teaching-Learning Process**
2. I deliver instruction through student-centered (SCL) approach.

□ **Extremely High** □ **Moderately High** □ **Low** □ **Very Low**

1. I align the teaching-learning activities and the intended learning outcomes.

□ **Extremely High** □ **Moderately High** □ **Low** □ **Very Low**

1. I align the teaching-learning activities and the assessment task.

□ **Extremely High** □ **Moderately High** □ **Low** □ **Very Low**

1. I align the teaching methods and strategies with the goals identified in the learning outcomes.

□ **Extremely High** □ **Moderately High** □ **Low** □ **Very Low**

1. I identify the teaching and learning activities that facilitate the achievement of course learning outcomes.

□ **Extremely High** □ **Moderately High** □ **Low** □ **Very Low**

1. I motivate the students’ understanding of the outcomes they are meant to achieve.

□ **Extremely High** □ **Moderately High** □ **Low** □ **Very Low**

1. I emphasize the knowledge and content (Cognitive domain) in the teaching and learning activities.

□ **Extremely High** □ **Moderately High** □ **Low** □ **Very Low**

1. I emphasize students’ skills and competencies (psycho-motor domain) in the teaching and learning activities.

□ **Extremely High** □ **Moderately High** □ **Low** □ **Very Low**

1. I emphasize the values and attitudes (affective domain) in the teaching-learning activities.

□ **Extremely High** □ **Moderately High** □ **Low** □ **Very Low**

1. I facilitate the learning activities for different types of learners in a diverse environment.

□ **Extremely High** □ **Moderately High** □ **Low** □ **Very Low**

*Section VI OBE Implementation in the Area of Assessment*

1. **Assessment**
2. I use different assessment tools to evaluate students’ progress.

□ **Extremely High** □ **Moderately High** □ **Low** □ **Very Low**

1. I assess students’ knowledge.

□ **Extremely High** □ **Moderately High** □ **Low** □ **Very Low**

1. I assess students’ skills and competencies.

□ **Extremely High** □ **Moderately High** □ **Low** □ **Very Low**

1. I assess students’ values and attitudes.

□ **Extremely High** □ **Moderately High** □ **Low** □ **Very Low**

1. I align the teaching methods and assessment methods.

□ **Extremely High** □ **Moderately High** □ **Low** □ **Very Low**

1. I align the assessment procedure and tools with the learning outcomes.

□ **Extremely High** □ **Moderately High** □ **Low** □ **Very Low**

1. I develop rubrics to assess the attainment of the institutional outcomes.

□ **Extremely High** □ **Moderately High** □ **Low** □ **Very Low**

1. I develop rubrics to assess the attainment of program outcomes.

□ **Extremely High** □ **Moderately High** □ **Low** □ **Very Low**

1. I develop rubrics to assess the attainment of course outcomes.

□ **Extremely High** □ **Moderately High** □ **Low** □ **Very Low**

1. I assess the level of students performance compared with the intended learning outcomes.

□ **Extremely High** □ **Moderately High** □ **Low** □ **Very Low**

*Section VII Effective Teaching and Learning Practices*

1. What teaching and learning strategies do you use in classroom? (You can select more than one option!)

□ Group Work

□ Lecture

□ Case Study

□ Demonstration

□ Field Trip

□ Simulations

□ Invite lecturers from industry

□ Experiments

□ Immersion

□ Research

*Section VIII Assessment Methods Used to Evaluate Outcomes*

1. What assessment method(s) do you use to evaluate the outcomes? (You can select more than one option!)

□ Written Examination

□ Assignments

□ Projects

□ Practical Examination

□ Portfolio

□ Use of Rubrics

□ Self-Evaluation

□ Peer Evaluation

□ Observations

□ Demonstrations

□ Journals
